# Early and late-onset nonconvulsive status epilepticus after stroke

**DOI:** 10.1590/0004-282X-ANP-2020-0018

**Published:** 2021-05-01

**Authors:** Eylem Özaydın Göksu, Fatma Genç, Nesrin Atiş, Yasemin Bıçer Gömceli

**Affiliations:** 1 Antalya Education and Research Hospital Department of Neurology Antalya Turkey Antalya Education and Research Hospital, Department of Neurology, Antalya, Turkey.; 2 Siirt State Hospital Siirt Turkey Siirt State Hospital, Siirt, Turkey.

**Keywords:** Stroke, Epilepsy, Status Epilepticus, Acidente Vascular Cerebral, Epilepsia, Estado Epiléptico

## Abstract

**Background:**

Nonconvulsive status epilepticus (NCSE) is a condition that needs timely diagnosis and treatment. It has insignificant clinical features and presents high risk of misdiagnosis.

**Objective:**

To investigate NCSE among patients with stroke, given that stroke plays an important role in the etiology of NCSE.

**Methods:**

In this retrospective study, acute stroke patients who were admitted and followed up at a stroke outpatient clinic between January 2013 and March 2016 were included. Patients with previous histories of epilepsy, brain tumor, head trauma, hypertensive encephalopathy, arteriovenous malformation, subarachnoid hemorrhage or cerebral venous thrombosis were excluded. Demographic properties, stroke etiology, imaging method, EEG findings, stroke severity according to the NIHSS score, functional disability and modified Rankin Scale were recorded for all patients.

**Results:**

Thirty-nine out of 792 stoke patients experienced NCSE. The mean age of the study population was 70±1.2 years (min-max: 46‒90). The study population was composed of 28 females (71.8%) and 11 males (28.2%). NCSE had early onset in 23 patients (59%) and late onset in 16 (41%). The early-onset NCSE patients were older and this was statistically significant between the groups (early onset: 73.5±11.5; late onset: 65.9±12.1; p=0.04). A history of previous stroke was more frequent in the late-onset NCSE group (14; 87,5%) than in the early-onset group (11; 47.8%) (p=0.01). The prognosis was worse in the early-onset group, but without statistical significance.

**Conclusion:**

Changes in mental status in the early stages of stroke are mostly attributed to stroke itself, but NCSE should be suspected in the right clinical setting, such as in older patients with suspicious anatomical and clinical associations.

## RESUMO

**Introdução:**

O Estado de Mal Não-Convulsivo (EMNC) é uma condição que necessita de diagnóstico e tratamento oportunos. Possui características clínicas insignificantes e apresenta alto risco de diagnóstico incorreto.

**Objetivo:**

Investigar o EMNC entre pacientes com acidente vascular cerebral (AVC), visto que ele desempenha um papel importante na etiologia do EMNC.

**Métodos:**

Neste estudo retrospectivo, pacientes com AVC agudo que foram admitidos e acompanhados em um ambulatório de AVC entre janeiro de 2013 e março de 2016 foram incluídos. Pacientes com história prévia de epilepsia, tumor cerebral, traumatismo cranioencefálico, encefalopatia hipertensiva, malformação arteriovenosa, hemorragia subaracnóidea ou trombose venosa cerebral foram excluídos. Propriedades demográficas, etiologia do AVC, método de imagem, achados de EEG, gravidade do AVC pela escala NIHSS, incapacidade funcional e escala de Rankin modificada foram registrados para todos os pacientes.

**Resultados:**

Um total de 39 dos 792 pacientes com AVC experimentaram EMNC. A idade média da população do estudo foi de 70±1,2 anos (mín-máx: 46‒90). A população do estudo foi composta por 28 mulheres (71,8%) e 11 homens (28,2%). O EMNC teve início precoce em 23 pacientes (59%) e início tardio em 16 (41%). Os pacientes com EMNC de início precoce eram mais velhos, o que foi estatisticamente significativo entre os grupos (início precoce: 73,5±11,5; início tardio: 65,9±12,1; p=0,04). Um histórico de AVC prévio foi mais frequente no grupo com EMNC de início tardio (14; 87,5%) do que no grupo de início precoce (11; 47,8%) (p=0,01). O prognóstico foi pior no grupo de início precoce, mas sem significância estatística.

**Conclusão:**

As alterações no estado mental nos estágios iniciais do AVC são atribuídas principalmente ao próprio AVC, mas deve-se suspeitar de EMNC com diagnóstico clínico correto, como em pacientes mais velhos com associações anatômicas e clínicas suspeitas.

## INTRODUCTION

Stroke is known to be a common cause of symptomatic epilepsy in older patients. Status epilepticus (SE) can present as the initial clinical reflection of an acute stroke^1^^,^^2^.

Nonconvulsive status epilepticus (NCSE) is characterized by altered mental status without convulsive motor activity^3^. In 1996, NCSE was defined by Kaplan as a state of nonconvulsive seizure with continuous or near-continuous epileptiform discharges lasting longer than 30 minutes^4^^,^^5^^,^^6^^,^^7^.

In 2015 Trinka et al. defined NCSE as an epileptic condition with reduced or altered consciousness and vegetative or merely subjective symptoms such as auras, but without major convulsive movements, lasting for at least 10 min^8^. The incidence of SE in the United States has been reported as 15‒20 patients per 100,000 population and NCSE accounts for 63% of this number^9^. In a prospective study, NCSE was detected in 26% of patients with altered mental status^10^. Although immediate diagnosis and treatment is crucial for NCSE, the diagnosis is often missed or confounded with a psychiatric disease, due to its subtle clinical features^7^^,^^11^. The etiology of NCSE shows considerable variability among age groups. Electrolyte imbalances, metabolic disarrangements, ischemic stroke, intracranial bleeding, brain tumor, traumatic brain injury, convulsive status epilepticus (CSE) and infections have all been implicated in the etiology of NCSE^12^. In a previous prospective study, stroke was the most frequent etiological reason for NCSE in intensive care patients^10^.

Early detection and treatment of NCSE is important, since increased blood flow to the brain during ictal activity, cytotoxic edema and cerebral vasoconstriction will cause cerebral ischemia and increased metabolic demand, which might lead to increased brain damage^13^^,^^14^^,^^15^.

In this study, we aimed to investigate the properties of patients with early and late-onset NCSE secondary to ischemic stroke.

## METHODS

In this retrospective study, acute stroke patients who were admitted and followed up at stroke outpatient clinics between January 2013 and March 2016 were included. Acute stroke was defined as presence of neurological signs and symptoms that were attributable to a specific vascular region, lasting more than 24 hours, and documented with CT/MRI within seven days of presentation.

The exclusion criteria comprised presence of the following: I- previous epilepsy; II- brain tumor; III- head trauma; IV- hypertensive encephalopathy; V- arteriovenous malformation; VI- subarachnoid hemorrhage; VII- cerebral venous thrombosis; VIII- history of dementia; IX- associated infections; or X- need for admission to an intensive care unit. In addition, patients who were found to have seizure activity secondary to alcohol withdrawal, psychotropic medication use or electrolyte disturbances were excluded.

**Figure 1:**
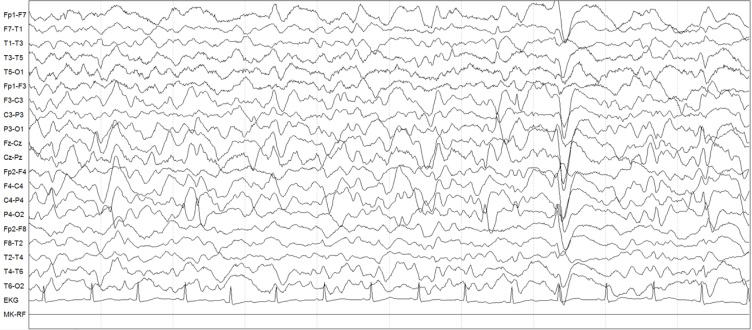
EEG recording of a 71-year-old female patient presenting with apathy. Electrodes were placed using the international 10–20 system of electrode placement. Low-frequency filter 1 Hz, high-frequency filter 70 Hz, sensitivity 7 UV. The recording shows nonconvulsive status epilepticus (NCSE) characterized by generalized bilateral periodic delta waves and sharp waves with a high amplitude (80–150 μV) at intervals from 0.5 to 1 s, which were identified as generalized periodic discharges. Although the patient was in a state of apathy without obvious motor signs, the findings were suggestive of a seizure involving her face or limbs.

The symptoms of all the patients were grouped as apathy, somnolence, fluctuation in consciousness, acute confusional state, behavioral disorder and automatism. EEG recordings were performed on the patients during hospitalization or outpatient visits to neurology outpatient clinics, if they presented an acute confusional state, apathy, delirium or mental status change. The standard placement of 10‒20 electrodes was used for the EEG recordings and the standard recording phase lasted 30 minutes. A neurologist and an EEG technician were both present during the EEG recording. When an ictal pattern was noticed, the patient was evaluated by a neurologist (Figure 1).

The diagnosis of NCSE was made in the presence of clinical and accompanying EEG findings. Clinically, NCSE was defined as epileptic seizures lasting more than 10 min and without prominent motor symptoms and/or convulsions. Clinical findings that were suggestive of NCSE included an acute confusional state, behavioral or cognitive change from baseline, apathy, drowsiness and mental fluctuations. We used the specific electrographic criteria for NCSE that were defined by Beniczky et al.^16^ (Table 1).

**Table 1:** Working clinical criteria for nonconvulsive status epilepticus.

**1. Patients without known epileptic encephalopathy**	
EDs > 2.5 Hz, or	
EDs ≤ 2.5 Hz or rhythmic delta/theta activity (> 0.5 Hz) and one of the following:	EEG and clinical improvement after IV AED^a^, or Subtle clinical ictal phenomena during the EEG patterns mentioned above, or Typical spatiotemporal evolution^b^
**2. Patients with known epileptic encephalopathy**	
Increase in prominence or frequency of the features mentioned above, in comparison with baseline, with observable change in clinical state	
Improvement of clinical and EEG^a^ features with IV AEDs	

Eds: epileptiform discharges (spikes, poly-spikes, sharp-waves, sharp-and-slow-wave complexes); IV AEDs: intravenous antiepileptic drugs.

a If EEG improvement occurs without clinical improvement, or if fluctuation without definite evolution, this should be considered as a possible NCSE.

b Incrementing onset (increase in voltage and change in frequency), or evolution in pattern (change in frequency > 1 Hz or change in location), or decrementing termination (voltage or frequency)^16^.

NCSE detected within 7 days of stoke was defined as early onset and after 7 days was defined as late onset. Patients were classified according to their stroke syndrome, reasoning and severity of condition, after neuroimaging, doppler ultrasound, echocardiography and ECG. Stroke etiology was classified according to the TOAST (Trial of ORG 10172 in Acute Stroke Treatment) criteria^17^.

The National Institutes of Health Stroke Scale (NIHSS) was used to assess the severity of stroke^18^. Demographic properties, etiology of stroke, neuroimaging methods used, EEG results and NIHSS of patients were recorded.

### Statistical analysis

The study data were analyzed using Statistical Package for the Social Sciences 16.0 for Windows (SPSS Inc., Chicago, Illinois, USA). Demographic and baseline characteristics were summarized as means±SD for continuous variables and as percentages of the group for categorical variables. Non-normally distributed data were presented as medians (with interquartile range). Normality of distribution was assessed using the Kolmogorov-Smirnov test. The Mann-Whitney U test and chi-square test were used to compare the early and late-onset seizure groups. Value of p<0.05 were accepted as statistically significant.

## RESULTS

Thirty-nine out of 792 stroke patients experienced NCSE between January 2013 and March 2016. The mean age of the study population was 70.0±1.2 years (min-max: 46-90). The study population was composed of 28 female patients (71.8%) and 11 male patients (28.2%). NCSE had early onset in 23 patients (59%) and late onset in 16 patients (41%). NCSE was detected during the hospital stay in four cases in the late-onset group, whereas it was detected during outpatient visits in the rest of the group. The early-onset NCSE patients were older than the late-onset group, and this was statistically significant (early onset: 73.5±11.5 years; late onset: 65.9±12.1 years; p=0.04). There was no statistical significance in the gender difference among the NCSE groups (p=0.3).

The most common clinical presentation was apathy (n=13; 33.3%). This was followed, in order of frequency, by somnolence (n=10; 25.6%), fluctuation in consciousness (n=7; 17.9%), acute confusional state (n=6; 15.4%), behavioral disorder (n=2; 5.1%) and automatism (n=1; 2.6%) (Table 2).

**Table 2:** Clinical presentation.

	Early-onset NCSE	Late-onset NCSE	
Apathy	6	7	13 (33.3%)
Somnolence	8	2	10 (25.6%)
Fluctuation in consciousness	5	2	7 (17.9%)
Confusional state	2	4	6 (15.4%)
Behavioral disorder	1	1	2 (5.1%)
Automatism	1	0	1 (2.6%)

Hypertension was the most frequent chronic disease in both groups. Histories of previous stroke were more frequent in the late-onset NCSE group: early onset: 11 (47.8%); late onset: 14 (87.5%); p=0,01. NCSE was secondary to ischemic stroke in 35 patients and hemorrhagic stroke in four patients. Intracranial hemorrhage was present in 4 patients: two of them in the early onset group had deep intracranial hemorrhage (ICH); and the other two patients had lobar hemorrhage.

According to the TOAST classification, in descending order, patients experienced I- cryptogenic stroke (n=18); II- cardioembolic stroke; III- large artery thrombosis (n=3); and small vessel thrombosis (n=3).

Although there was no statistically significant difference, the prognosis seemed to be worse in the early-onset group (Table 3).

**Table 3:** Demographic properties.

		Early-onset NCSE	Late-onset NCSE	p value
Age (mean±SD)		73.5±11	65.1±12	0.05 (Student t test)
Male sex (%)		21.7	37.5	0.30 (Fisher's exact test)
HT (%)		73.9	93.8	0.20 (Fisher's exact test)
DM (%)		30.4	43.8	0.39 (Chi-square test)
Previous stroke (%)		47.8	87.5	0.01 (Chi-square test)
AF		21.7	25	1 (Fisher's exact test)
TOAST classification				
	Cardioembolic (%) Large vessel (%) Small vessel (%) Cryptogenic (%)	35 5 - 60	26.7 13.3 20 40	
Stroke type (ischemic /hemorrhagic)		20/3	15/1	0.72 (Mann-Whitney U test)
Lesion location (%)				
	MCA PCA Brain stem White matter signal hyperintensities	75 20 5 -	66.7 13.3 6.7 13.3	
mRS (median-IQR)		3 (2-3)	3 (1.5-3)	0.83 (Mann-Whitney U test)
NIHSS (median-IQR)		8 (6-12)	6.5 (5.25-6.5)	0.45 (Mann-Whitney U test)
Previous epilepsy (%)		21.7	37.5	0.30 (Fisher's exact test)
Death (%)		26.1	6.3	0.20 (Fisher's exact test)

SD: Standard deviation, HT: Hypertension, DM: Diabetes Mellitus, AF: Atrial Fibrillation, TOAST: Trial of ORG 10172 in Acute Stroke Treatment, MCA: Middle Cerebral Artery, PCA: Posterior Cerebral Artery, mRS: modified Rankin scale, NIHSS: National Institute of Health Stroke Score.

## DISCUSSION

According to the results from this study, 4.93% of all the stroke patients experienced NCSE. The patients in the early-onset NCSE group had experienced fewer previous strokes and they were older than those in the late-onset NCSE group.

Reports in the literature regarding the frequency of seizure after stroke and its progression to epilepsy have indicated great variability. There are few observational studies on NCSE after stroke. A prospective study by Belcastro et al. reported that the frequency of NCSE was 3.6%, and that 62% of their population had early-onset NCSE^19^. Our results were concordant with their study in terms of frequency of NCSE and time of onset after stroke. In our study, 59% of the patients were in the early-onset NCSE group.

In previous studies, NCSE frequency became higher with older age and female sex^20^^,^^21^. In another study, NCSE was reported to be 4 to 43 per 100,000 in the older patient population, while this number was 1.5 per 100.000 for all the age groups^20^. In yet another study, NCSE was more likely to be detected in older females, but without a clear reason^21^.

Our study population was older, with a mean age of 70 years (min-max: 46‒90). The early-onset NCSE group was older and this was statistically significant (early onset: 73.5±11.5 years; late onset: 65.9±12.1; p=0.04). Although there was no statistically significant sex difference regarding the time of onset between the groups, 71% of the study population was female, which supported the findings of previous studies.

NCSE can cause a variety of symptoms, such as somnolence and cognitive dysfunction. Patients with NCSE may be diagnosed as presenting a physiatric illness because of the nonspecific and subtle features of the clinical presentation. The signs and symptoms of NCSE have a highly variable spectrum, from minor mood disturbance to delirium and coma. In our cohort, apathy was the most common presenting symptom and following apathy, somnolence, fluctuation in consciousness, acute confusional state, behavioral disorder and automatism were the other symptoms of presentation^7^^,^^22^.

The association between stroke etiology and SE (status epilepticus) is inconsistent. In the study by Velioğlu et al., they were unable to detect any association between the etiology of stroke and localization. However, in the study by Belcastro et al., it was reported that large-vessel atherothrombosis is indicative of NCSE^19^^,^^23^.

Also in the study by Belcastro et al, the median NIHSS score after stroke was 13 (9‒15) in the NCSE group, while the median NIHSS score was 3 (3‒7) for the non-NCSE group. The median NIHSS score in our study was lower than what was reported by Belcastro et al. However it was higher than the score for the non-NCSE group in their study. This suggests that there is higher frequency of NCSE among patients with higher NIHSS scores^19^.

Post-stroke seizures (PSS) may present with altered mental status alone, thus leading to delay in evaluation and treatment. Epileptic seizures, on the other hand, increase the metabolic demand of the ischemic tissue surrounding the infarct, and may negatively impact the overall recovery of these patients^24^.

Some previous studies have reported higher levels of mortality and morbidity with NCSE after stroke. On the other hand, some other reports have claimed that NCSE is a benign condition and does not need aggressive treatment^20^^,^^25^^,^^26^. One previous study indicated that early appropriate treatment was associated with favorable prognosis for patients with NCSE^7^^,^^27^. A recently published prospective study showed that NCSE had a poor prognosis, with a mortality rate of 31%. Occurrence at younger age and prolonged NCSE have been shown to have statistically significant higher mortality rates^10^. Although we could not detect any statistically significant difference, the prognosis was worse in the early-onset NCSE group. The reason for higher mortality in the early-onset group may be secondary to older age and the effects of stroke itself in the early period.

Stroke in itself seems to be associated with increased risk of NCSE. All types of ischemia, i.e. not only cortical but also lacunar infarcts, have the possibility of developing subsequent NCSE. When deep ICHs are compared with lobar ICHs including an insular area, patients tend to develop NCSE more frequently with a deep hematoma location^28^. In our study, we had a total of 4 patients with intracranial hemorrhage: two of the patients in the early onset group had deep ICH and two other patients had lobar hemorrhage.

Vespa et al. reported that the presence of NCSE was associated with increased mortality and morbidity among patients with intracerebral hemorrhage. However, in our study we could not detect such a result despite having a high number of intracerebral hemorrhage patients in the early period^29^.

The present study has some limitations. It was a retrospective single-centered study. Thus, the small study population may not have led us to precise conclusions regarding NCSE. However, our study is unique in terms of comparing early and late-onset NCSE patients after stroke. Previous studies compared patients with and without NCSE, or focused either on early or on late-onset NCSE after stroke.

In conclusion, NCSE after stroke is not an uncommon clinical entity. Changes in mental status in the early stages of stroke are most probably attributed to stroke, but NCSE should be suspected in the right clinical setting, especially among older patients who have suspicious anatomical and clinical associations.
